# Loop-Mediated Isothermal Amplification (LAMP) Assay to Detect Toxoplasmosis in Schizophrenia Patients

**DOI:** 10.18502/ijpa.v15i3.4193

**Published:** 2020

**Authors:** Hadi MIRAHMADI, Raheleh HASANZADEH, Hamid MALEK RAEESI, Shirzad FALLAHI, Mahdi KHOSHSIMA SHAHRAKI, Alireza BADIRZADEH

**Affiliations:** 1. Infectious Diseases and Tropical Medicine Research Center, Resistant Tubercluosis Institute, Zahedan University of Medical Sciences, Zahedan, Iran; 2. Department of Parasitology and Mycology, Faculty of Medicine, Zahedan University of Medical Sciences, Zahedan, Iran; 3. Department of Parasitology and Mycology, Faculty of Medicine, Lorestan University of Medical Sciences, Khorramabad, Iran; 4. Department of Medical Parasitology, Zabol University of Medical Sciences, Zabol, Iran; 5. Department of Parasitology and Mycology, School of Medicine, Iran University of Medical Sciences, Tehran, Iran

**Keywords:** Loop-mediated isothermal amplification (LAMP), *Toxoplasma gondii*, Toxoplasmosis, Schizophrenia

## Abstract

**Background:**

*Toxoplasma gondii* (*T. gondii*) causes an important parasitic infection known as toxoplasmosis, which is a globally distributed important zoonosis. One of the major serious characteristics of *T. gondii* is its ability to manipulate the behavior of intermediate hosts. We performed a cross-sectional study to determine toxoplasmosis in schizophrenic patients, as one of the major neuropsychiatric disorders, using loop-mediated isothermal amplification (LAMP) technic by targeting parasite B1 gene.

**Methods:**

Blood samples were taken from 118 schizophrenic patients hospitalized in tow hospitals including Baharan, Clinic of Psychiatric Ali-ibn-Abi-Talib Hospital (in Zahedan City), and Amir-al Momenin Psychiatric Hospital (in Zabol City), Sistan and Baluchestan Province, southeast Iran in 2016. They were analyzed using LAMP, and compared with the previous data of nested-PCR and serology.

**Results:**

Out of the 118 schizophrenic individuals, 56 patients (47.4%) were found to be infected with *T. gondii*. The diagnosis of toxoplasmosis was confirmed in 41 patients (34.7%) via the nested-PCR. The seroprevalence of toxoplasmosis in schizophrenic patients was 55.9% (66/118).

**Conclusion:**

We found a high efficiency of LAMP method in identifying toxoplasmosis and its high prevalence among schizophrenic patients. Our findings could provide viable offer implications for the prevention of schizophrenia.

## Introduction

With uncertain causes and a very high global prevalence, schizophrenia as a neurological disorder has harmful impacts upon affected patients and their relatives (1, 2). The disease inflicts both direct and indirect costs on human society in terms of the real resources concerning medical service activities, lost work productivity, and associated socioeconomic difficulties (2). According to the literature, the main cause of its development is commonly unknown; however, various scientists assume that causes may include both genetic and environmental factors. Among those factors, one of the main etiological agents is being infected with infectious pathogens such as intracellular parasite *Toxoplasma gondii* (*T. gondii*) which is related to the parasite neurotropism and its effects on brain dysfunction (3, 4).

*T. gondii*, the causative agent of toxoplasmosis, is a coccidian protozoan parasite of the apicomplexan family, and one of the major zoonotic disease whose life cycle can be fulfilled solely in the felids (cats) as the final definitive hosts (5–7). Warm-blooded animals such as humans act as intermediate hosts. *Toxoplasma* parasite infects almost 30% of the world’s human population in all countries (1, 8). Major risk factors for being infected with this protozoan parasite in humans’ society are the use of inadequately cooked and/or raw meat containing tissue cysts of *Toxoplasma*, ingest of sporulated oocysts from different sources such as contaminated water or soil, and the vertical transmission of parasite via the placenta (8).

Different studies have been carried out to describe the linkage between *T. gondii* infection and schizophrenia and other mental disorders (4, 9). Although several studies have elucidated that schizophrenic patients have an increased seroprevalence of anti-*Toxoplasma* IgG and IgM antibodies (3, 4), other studies have failed to show a significant connection between developing schizophrenia and toxoplasmosis (10–12). Various recent experiments with contradictory findings in distinct parts of Iran have been performed to evaluate the association of schizophrenia with toxoplasmosis (1, 13–15). Furthermore, studies in animal models have demonstrated that toxoplasmosis can manipulate the behavior in mice, increase the chances when a mouse is hunted and eaten by a cat, and consequently enable the *Toxoplasma* parasite to fulfil its complicated life cycle (16).

LAMP method is a novel and user-friendly DNA amplification technic with a very high efficiency, sensitivity, simplicity, and specificity under isothermal conditions (17, 18).

This technic has already been successfully implemented for rapid identification and/or detection of several parasitic pathogens including *Schistosoma* spp. (19), *Fasciola* spp. (20) *Babesia* spp. (21), *Trypanosoma* spp. (22), *Cryptosporidium* spp. (23), *Entamoeba histolytica* (24), *Plasmodium* spp. (25) and *Toxoplasma* (18, 26).

Due to lack of any documented report on the possible link between *T. gondii* infection and schizophrenia in Sistan and Baluchestan province (southeast Iran) and more importantly its molecular identification in this region, the present study aimed to investigate the molecular diagnosis of toxoplasmosis in schizophrenic individuals using LAMP method and targeting the repetitive conserved region of B1 gene in *T. gondii* via comparing it with the previously published data

## Materials and Methods

### Study population

**Fig. 1:**
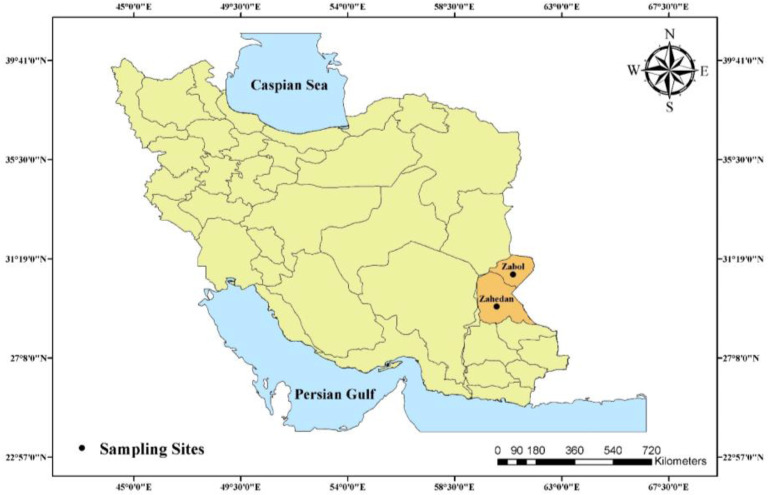
Location of sampling sites (Zabol and Zahedan cities) in Sistan and Baluchestan Province, Southeast of Iran (Created by Arc GIS version 10.2)

In the present cross-sectional study, a total of 118 confirmed schizophrenic individuals who referred to distinct hospitals (Baharan, Clinic of Psychiatric Ali-ibn-Abi-Talib hospital in Zahedan city, and Amir-al Momenin Psychiatric hospital in Zabol City) in two main cites of Sistan and Baluchestan Province, southeast Iran including Zahedan and Zabol were enrolled (Fig. 1). The study was done within a six-month period from Jun to Nov in 2016. The main inclusion criteria in the present study were: 1) schizophrenic patients who were chosen under the supervision of neurology consultants of Zahedan University of Medical Sciences, Zahedan, Iran (based on the Structured Clinical Interview for Diagnostic and Statistical Manual of Mental Disorders, fourth edition (DSM-IV-TR)) (27); 2) the individuals who aged above 18 yr; 3) individuals who volunteered to take part and signed written informed consent questionnaire before participating in the study.

Ethical approval was obtained from the Ethical Committee of Zahedan University of Medical Sciences, Zahedan, Iran (ethical code: IR.ZAUMS.REC.1395.14).

### Sample collection

From each schizophrenic patient who fulfilled the above-mentioned inclusion criteria, 4 ml whole blood in total was taken. All the samples under the cold-chain storage conditions were immediately transferred to the Department of Medical Parasitology, Zahedan University of Medical Sciences, Zahedan, Iran. These samples were centrifuged for 5 min at 4000 rpm. Then, sera were isolated to examine the specific anti-*Toxoplasma* IgM and IgG antibodies ELISA, as already described (28). Moreover, buffy coat extractions for molecular evaluations were performed for all samples. Both separated sera and buffy coats were separately poured in sterile 2 ml tubes and then were kept at −20 °C until they were used.

### DNA extraction and Nested-PCR

The purification of genomic DNA from buffy coat samples was done using the commercial DNA extraction kit [DynaBio Tm Blood/Tissue DNA Extraction MiniKit (Takapoozist, Iran)] according to the manufacturer’s guidelines, as described previously (28). Nested-PCR was done for molecular identifications via B1 gene, according to the procedures were described in our previous publication (28, 29).

### LAMP assay

As shown in Table 1, four main *T. gondii*specific primers targeting six highly conserved and repeated regions of the B1 gene (AF179871.1) were utilized for the LAMP identification assay, as previously described elsewhere (30). Briefly, the LAMP amplification was conducted in 25 μl of reaction mixtures including 40 pmol of each inner primers (FIP and BIP), 5 pmol of each outer primer (F3 and B3), 8 U of Bst DNA polymerase (New England Biolabs, USA), 1.4 mmol/μl of dNTP, 1X reaction buffer (Sigma-Aldrich), 6.5 μl of distilled water (DW), and 1 μl of purified template DNA. After adding 1 μl of fluorescent detection dye, the reaction mixture was incubated at 66 °C for 60 min and heated at 95 °C for 2 min to inactivate the DNA Polymerase. Both positive (purified DNA of *T. gondii* RH-strain) and negative controls (DDW) were added in each run of the assay.

**Table 1:** Nucleotide sequences of LAMP primers used for *Toxoplasma* identification in patients with schizophrenia

***Molecular Technique***	***Target gene***	***Sequence (5′-3′)***
LAMP	B1	BIP-TCGCAACGGAGTTCTTCCCAGTTTTGGCCTGATATTACGACGGAC FIP-TGACGCCTTTAGCACATCTGGT TTTTGATGCTCAAAGTCGACCGC F3-GGGAGCAAGAGTTGGGACTA B3-CAGACAGCGAACAGAACAGA

### Statistical analysis

In the present study, the data was analyzed using Graph-Pad Prism 6.0 for Windows (Graph-Pad Prism, San Diego, California, USA). The *P*-values less than 0.05 (*P*<0.05) were considered statistically significant.

## Results

**Fig. 2:**
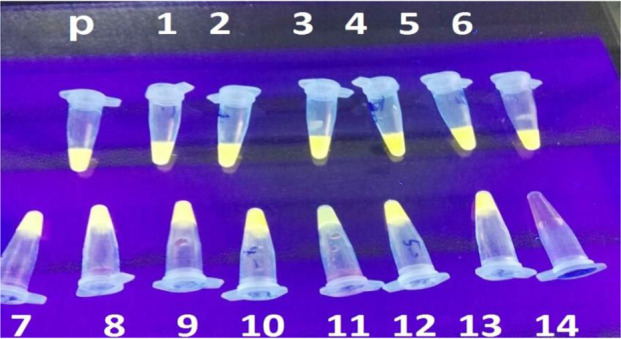
LAMP monitor of *T. gondii* DNA in schizophrenic patients. P: positive control, N: negative control; Tubes 1–14 represent patients samples

**Fig. 3:**
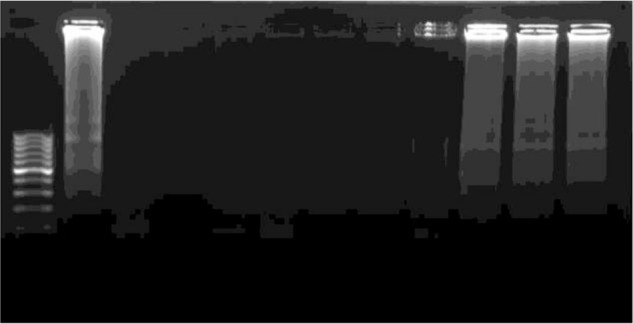
Agarose gel analysis of LAMP assay for the specific detection of *T. gondii* DNA based on B1 gene amplification. Lane 1: 100 bp molecular weight marker; Lane 2: positive control; Lane 3: negative control; lanes 4–9: negative test group from patients; lanes 10–12: positive test group from patients

Overall 118 individuals with confirmed schizophrenia and inclusion criteria, who lived in Zahedan and Zabol (Fig. 1), were recruited for the present study. Out of the 118 confirmed schizophrenic patients, 19 were female (16.1%) and 99 were male (83.9%), with a median age of 38 yr (range: 19 to 55 yr). The diagnosis of toxoplasmosis was confirmed in 41 patients (34.7%) using the nested-PCR. The results of our study using LAMP assay showed that, out of the 118 schizophrenic individuals, 56 patients (47.4%) were found to be infected with *T. gondii* (Figs. 2 and 3). The comparison between LAMP and nested-PCR results, which was described our previous publication, showed that the efficiency of the former was higher than that of the latter one (*P* < 0.05) (Table 2). The serology data in our previous work showed that the seroprevalence of toxoplasmosis in schizophrenic patients was 55.9% (66/118). Distinct titers of the anti-*Toxoplasma* IgG were found in 48 cases (40.67%), while the anti-*Toxoplasma* IgM and IgG/IgM were positive in 4 cases (3.37%) and 14 cases (11.86%), respectively (Table 2).

**Table 2:** Comparative results of Nested-PCR and LAMP techniques in *Toxoplasma*-seropositive schizophrenic patients based on B1 gene

***Diagnostic methods***	***ELISA***				***Total of 66/118 seropositive***
	***IgG+/IgM+***	***IgG-/IgM-***	***IgG-/IgM+***	***IgG+/IgM-***	
Nested-PCR	12 (86%)	2 (3%)	4 (100%)	25 (52%)	43 (62%)
LAMP	14(100%)	3 (4.5%)	4 (100%)	35 (73%)	56 (85%)

## Discussion

Since the 1950s, hidden association between toxoplasmosis, as a globally important parasitic disease, and schizophrenia, as one of the major neuropsychiatric disorders, has been investigated (2, 9). In the mid-1990s, the infectious hypothesis of schizophrenia development via close contact with cats shed light on the plausible roles of *T. gondii* in this mental disorder (31). The prevalence of toxoplasmosis in schizophrenic patients is very high, and the parasitic pathogen increases the risk of getting schizophrenia (3, 4, 32). Furthermore, a study in Lorestan Province (Western Iran) have shown that psychiatric individuals such as bipolar and schizophrenia cases had a remarkably higher frequency of *T. gondii* infection than healthy patients (33).

In the present study, for the first time, the prevalence rate of toxoplasmosis in confirmed schizophrenic patients was investigated using LAMP method and was compared with the serology and molecular data of our previous publication in Southeastern Iran (28). We found that 47.4%, 34.7%, and 55.9% of schizophrenic patients were positive for *T. gondii* via LAMP, nested-PCR, and serology, respectively. Furthermore, distinct titers of the anti-*Toxoplasma* IgG were found in 48 cases (40.67%) which represented the chronic phase of disease in the schizophrenic patients. In addition, the anti-*Toxoplasma* IgM was positive in 4 cases (3.37%) revealing the existence of a relationship with the acute phase of the infection. Due to the mainly hot and dry climate in the Southeast of Iran, the prevalence of *T. gondii* was lower than it was in other regions of the country. In this regard, different studies in the Southeast of Iran showed that the prevalence rates of *T. gondii* in different human groups such as general population, pregnant women, and healthy blood donors were 22.8%, 25%, and 22.7%, respectively (15, 34–36). Various studies have elucidated that environmental conditions such as different climates are very important for *Toxoplasma* oocysts survival; therefore, the possible explanation for this phenomenon can be the reduction of parasite oocysts due to hot and dry climates (37, 38).

Distinct molecular assessments based on several targets and methods have been investigated and reported for the diagnosis of *Toxoplasma* parasite (26). A rapid screening of the individuals infected by *T. gondii* dispensed extremely remarkable information for the surveillance of parasite control and the precise prevalence estimation of the disease in the endemic regions of the country (18, 26). In this respect, we used an exceedingly sensitive LAMP technic targeting the B1 gene to diagnose rapid identification of *T. gondii* DNA in the schizophrenic patients (18, 26). Molecular markers that target the genes such as B1 and conserve the repeated region of the *Toxoplasma* genome show excellent sensitivity and specificity for isolated clinical samples; therefore, genomic target of B1 was chosen in the present study (18). Our data elucidated that the LAMP method was highly specific for the identification of the *T. gondii*, providing more evidence for the other studies (18, 26, 39).

The LAMP assay has higher diagnostic sensitivity due to the 4–6 primers utilized in the current reaction which target 6–8 different internal regions on the B1 gene as the target DNA (18). Owing to the isothermal conditions in this simple method and less specialized tools, the amplification of DNA can be gained via ordinary incubators including block heater and/or water bath; therefore, this assay can be utilized to detect toxoplasmosis in the field study. Furthermore, one of the main advantages of the LAMP is that the amplification of DNA can be quickly investigated through visual inspection. The LAMP assay, as a fast and feasible field diagnostic equipment, was validated in the schizophrenic patients infected by *T. gondii*. LAMP assay is very efficient and sensitive in the infected patients of distinct parasitic diseases such as leishmaniasis and toxoplasmosis (26, 40).

The results of the present study should be interpreted considering several limitations. First, there was no control groups (healthy individuals) to evaluate plausible associations between the diseases. Second, no specific questionnaire was designed to compare the related risk factors such as contact with cats and sources of drinking water between toxoplasmosis and schizophrenia.

## Conclusion

Our findings in the present and previous studies demonstrated a high prevalence of toxoplasmosis in schizophrenic patients; hence, due to simplicity and sensitivity of LAMP assay, it is recommended as a suitable technic for the regular molecular detection of *T. gondii* DNA.

## Ethical considerations

Ethical issues (Including plagiarism, informed consent, misconduct, data fabrication and/or falsification, double publication and/or submission, redundancy, etc.) have been completely observed by the authors.
